# A Multiple Stakeholder Perspective on the Drivers and Barriers for the Implementation of Lifestyle Monitoring Using Infrared Sensors to Record Movements for Vulnerable Older Adults Living Alone at Home: A Qualitative Study

**DOI:** 10.3390/ijerph19010570

**Published:** 2022-01-05

**Authors:** Anna M. Braspenning, Karlijn Cranen, Liselore J. A. E. Snaphaan, Eveline J. M. Wouters

**Affiliations:** 1 Tranzo, Tilburg School of Social and Behavioral Sciences, Tilburg University, 5037 DB Tilburg, The Netherlands; k.cranen@gmail.com (K.C.); liselore.snaphaan@GGZE.nl (L.J.A.E.S.); e.j.m.wouters@uvt.nl (E.J.M.W.); 2 School of Allied Health Professions, Fontys University of Applied Science, 5631 BN Eindhoven, The Netherlands; 3 Research Unit Evidence Based Management of Innovation, Mental Health Care Institute Eindhoven, 5626 ND Eindhoven, The Netherlands

**Keywords:** lifestyle monitoring, dementia, independent living, technology implementation, older adults, community dwelling, normalization process theory

## Abstract

A variety of technologies classified as lifestyle monitoring (LM) allows, by unobtrusive monitoring, for supporting of living alone at home of vulnerable older adults, especially persons with neurocognitive disorders such as dementia. It can detect health deterioration, facilitate early intervention, and possibly help people avoid hospital admission. However, for LM to redeem its intended effects, it is important to be adopted by involved stakeholders such as informal and formal caregivers and care managers. Therefore, the aim of this qualitative study is to understand factors that drive or impede successful implementation of LM for vulnerable older adults, specifically using infrared sensors to record movements, studied from a multiple stakeholder perspective. An open coding process was used to identify key themes of the implementation process. Data were arranged according to a thematic framework based on the normalization process theory (NPT). All stakeholders agreed that LM could lead to various health benefits for older adults using LM. However, some did not perceive the LM system to be cost-efficient and expressed a need for more flexible health care structures for LM to be successfully implemented. All stakeholders acknowledged the fact that LM requires a transition of care and responsibilities, a clear eligibility strategy for clients, and a clear ambassador strategy for health care professionals, as well as reliable technology. This study highlights the complex nature of implementing LM and suggests the need for alignment within constructs of the NPT among stakeholders about new ways of collaboration in supporting living alone at home.

## 1. Introduction

The demography in the world is changing and brings challenges for societies and health care systems; the need of care among older adults is increasing due to increased longevity. This demand of care has to be provided by the social environment, e.g., informal caregivers or by professional caregivers, while at the same time there is an increasing shortage of care personnel [1]. Cognitive decline and dementia are the main cause of disability amongst older adults [2]. In 2019, the number of people living with dementia was estimated to be around 50 million and is projected to increase to 150 million by 2050 [3].

To deal with mental health consequences, like dementia, there is an increased interest in facilitating the independence and autonomy of vulnerable older people at home with assistive technology or telecare in order to stay at home longer [4,5,6]. Assistive technologies or telecare consist of different combinations of single devices, e.g., fall detection systems, smart home systems, or lifestyle monitoring (LM). More specifically, LM is intended to record daily activity patterns by tracking important safety parameters of people living at home alone by sensors, such as infrared sensors recording movement. Sensors including AI technologies are one of the latest innovations to LM [7,8,9]. By thoughtfully doing so, LM aims for early diagnosis and intervention and possibly avoid hospital admission.

In spite of the potential of LM, it has not widely been adopted [10,11]. Implementation of technologies are often based on individual perspectives and trade-offs between benefits and consequences. Several small-scale studies have been conducted on LM mostly investigating effectiveness or individual perspectives on usability [12,13,14]. For LM to redeem its intended effects, it is important that it is adopted by involved stakeholders and fits into the care processes of involved stakeholders, e.g., informal caregivers, professional caregivers, and managers of health care organizations. Stakeholders in the care process showed different perspectives on LM; informal caregivers and older adults showed perceived access delegation and flexibility in receiving alerts as important to tailor the technology for use, influencing their adoption [15], whereas the professional caregivers’ adoption depends on their involvement in the design and deployment process and the reliability and accuracy of the technology [16]. It is important that involved stakeholders communicate with each other in order to understand and act on the mutual and different perspectives on needs, roles, and knowledge and to achieve a common goal in implementation of LM starting with informing future users about LM [17].

What is lacking in the literature is sufficient theory-driven knowledge to introduce assistive technologies, which aim to facilitate successful implementation [18]. A theory that describes the process underlying implementation of interventions in dementia care, is the normalization process theory (NPT) [19]. The NPT derived from the normalization process model that was originally developed to understand how new interventions in health care become normalized [20]. NPT provides tools to understand the social processes of thinking, enacting, and organizing work to implement and adopt interventions in care processes within health care organizations. The NPT distinguishes four constructs: (a) coherence, (b) cognitive participation, (c) collective action, and (d) reflexive monitoring. The constructs help to account for how involved people understand and make sense of a new intervention and work they have to go through in order to succeed in the routine embedding of the new intervention (i.e., coherence), the work they have to do to enroll people to engage and participate with the new intervention (i.e., cognitive participation), the work they need to do to enact the new intervention (i.e., collective action), and reflect or appraise its effects (i.e., reflexive monitoring). The NPT can be useful to get detailed insights in stakeholders’ perspectives in different phases of implementation using the constructs of NPT and to unravel the complexity of implementation [21,22]. In addition, NPT can be useful because a shift in roles of involved stakeholders in the context of the care process while working with LM is expected. NPT helps to understand the roles among different stakeholders in a specific context of care [23,24]. The aim of this study is to understand factors that drive or impede successful implementation of LM for vulnerable older adults, specifically using infrared sensors to record movements, seen as important from a multiple stakeholder perspective.

## 2. Materials and Methods

### 2.1. Setting

In this study, LM was promoted through financial aid of the local government of the municipality of Breda (Breda, The Netherlands) and was freely accessible for vulnerable older adults, e.g., persons with dementia, living alone at home (sampling period 1 January 2018–31 December 2019). Information about LM was sent by the municipality of Breda by post to all older adults living alone at home. Costs for using LM were paid by the municipality of Breda. 

The LM system in this study uses passive infrared sensors to record movements of people in their homes. The majority of the sensors of the system investigated in this study are attached to the wall at about 1.40 m height at spots frequently used in the home where both daytime and night activity takes place (e.g., living room, bathroom, bedroom, refrigerator), as well as at the front and back door. The wireless sensor network of passive infrared sensors communicates with software in a cloud. Due to a self-learning algorithm, after creating a baseline lifestyle pattern in two weeks of use, the system configuration can provide useful information about the lifestyle of the users, e.g., presence in or out of bed, kitchen and toilet activity, level of activity, et cetera. Information from the sensors was collected and presented to informal caregivers through a platform, accessible by tablets or smartphones. Informal caregivers followed the activities of the older adult and received alerts when unusual behavior was detected.

Actors who played a role in the implementation were informal caregivers, health care professionals, and health care managers as the local government of the municipality of Breda decided to give them an active role in the implementation. 

The primary users of the LM system were informal caregivers of vulnerable older people living alone at home, as they were the persons who received alerts from the LM system. The health care professional had the role to support the use of the LM system to older adults living alone at home, in case people, for instance, those with MCI or dementia, needed care from a health care organization. The activities of the health care professional included providing information about the LM system to older adults and their informal caregivers. The health care managers decided who were the health care professionals in the health care organization to fulfill that supporting role. Finally, the system was installed by the LM company in the homes of vulnerable older adults after agreement to start with LM. 

### 2.2. Recruitment

A convenience sample of 14 participants was selected between September 2019 and March 2020 from the municipality of Breda. This study focused on the stakeholders who had a role in the health care process of the actual implementation of LM to the client and their informal caregivers. Therefore, we included a balance of three stakeholder groups involved in the implementation process of LM, namely informal caregivers, health care professionals, and health care managers who were initiators of LM in a health care organization. Interviews took place at the homes or workplaces of respondents. 

The study was approved by METC Brabant. Participants were informed of the procedure and purpose of the interview as well as the voluntary nature of their participation and confidentially was guaranteed. All participants willing to participate signed an informed consent form. 

### 2.3. Data Collection

In this qualitative case study, we used the NPT to help understand the contribution of factors of involved stakeholders that inhibit or enable the successful implementation of LM among older adults living home alone. According to NPT, “coherence”, “cognitive participation”, “collective action”, and “reflexive monitoring” are important constructs underlying eHealth implementation [25].

Based on NPT constructs, an interview guide was developed. The interview guide explored the perceived drivers and barriers throughout the implementation process of LM. Although areas for exploration were defined, the semistructured interview allowed for flexibility and deeper examination of issues arising. Interviews were conducted by KC (communication scientist) and IB (health scientist), lasted between ½ and 1 ½ h, and were guided by a semistructured interview guide (Appendix A).

### 2.4. Analysis

The audio of interviews was recorded and transcribed verbatim with participants’ permission. First, two coders (KC and AB) separately read all transcripts to familiarize themselves with the data. Data were arranged according to a thematic framework based on NPT constructs and a selection of topics evolving among informal caregivers, health care professionals, and managers was made. Next, the data for each NPT component was analyzed and arranged into subthemes using an inductive process, meaning that patterns, themes, and categories arise from the data [26]. The software Atlas.ti and Excel was used to conduct this process. Differences were discussed and resolved during discussion meetings. The credibility of the analysis was aided by ongoing discussion with two additional reviewers EW (medical doctor) and LS (neuroscientist), both having experience with qualitative analysis. To ensure confidentiality, we removed all identifying information from the quotes.

## 3. Results

### 3.1. Study Population

The research sample consisted of 14 respondents, 11 of whom were female. We included five informal caregivers, five health care professionals, and four health care managers. The health care professionals consisted of nurses and professional caregivers who worked in the home support, e.g., providing aid in cleaning the house. Participants’ age ranged from 32 to 69 years (Table 1).

### 3.2. General Interview Results

In this study, differences within the four constructs were found based on different interpretations of the constructs by different stakeholders, with conflicting results at some points. We found drivers and barriers for implementation within the constructs coherence, cognitive participation, collective action, and reflexive monitoring. In this study, there were limited results on reflexive monitoring; this step of evaluating LM and following up the use of LM was not embedded in the health care organizations or among informal caregivers. Table 2 shows the presence of drivers and barriers within the constructs of NPT for all stakeholders.

### 3.3. Reflection on NPT Construct Coherence 

In this construct, perceived values of LM and clarity and perceived values of (possible) roles are reflected. A driver for implementation regarding values of LM in this construct was that all involved stakeholders believe in the benefits of LM and the goal of LM. In this study, barriers were found in terms of absence of a clear cost efficiency of LM perceived by health care professionals and health care managers and lacking possibilities for flexibility of the current health care system, e.g., care hours need to be flexible, as perceived by informal caregivers and health care managers. As a result of these barriers, no clarity of roles was present.

**Table 2:** Drivers and barriers for implementation within constructs of NPT derived from two or more stakeholder groups.

Themes	Stakeholders		
	IC	HCP	HCM
**Coherence: in order to promote or inhibit the routine embedding of a practice.**			
Perceived health benefits of LM.	D	D	D
Unclear cost efficiency for the health care organization.	-	B	B
Lacking opportunities for flexibility of the health care system.	B	-	B
**Cognitive participation: to enroll individuals to engage with the new practice.**			
Lacking opportunities in transition of care and responsibilities.	B	B	B
**Collective action: to enact the new practice.**			
Unclear eligibility strategy for clients.	B	B	B
Unclear ambassador strategy (for HCP’s).	B	B	B
**Reflexive monitoring: to reflect on the new practice.**	B	B	B
Lacking perceived reliability of the technology.	B	B	B

IC = Informal caregiver, HCP = health care professional, HCM = health care manager, LM = lifestyle monitoring, NPT = normalization process theory, - not mentioned as driver or barrier, D = perceived driver experienced by stakeholder in this study, B = perceived barrier experienced by stakeholder in this study.

- *Perceived health benefits of LM—driver*

Results demonstrated that, in general, the different stakeholders held communal ideas of the expected benefits of LM and its aim. Drivers for acceptance of LM and successful implementation that were valued by all stakeholders were: an increased insight into the health status and daily living pattern of older adults living alone without the use of a camera, an increased independence of older adults, ultimately facilitating living at home longer, and a sense of safety through fall detection and the possibility to monitor a person without intruding privacy.

*“What our employees react on when it made sense in their eyes, that is mainly on the part of the vulnerability in which the day–night rhythm is of course very important.”*

(respondent 1, health care manager).

- *Unclear cost efficiency for the health care organization—barrier*

It is unclear for health care professionals and health care managers how they would financially benefit from LM and as such why they need to spend time on the introduction of LM to health care consumers. Health care professionals and health care managers felt that being able to substantiate decisions in care by upcoming problems measured by LM, e.g., a possible bladder infection, would be a driver for implementation. However, health care professionals felt that the technology company would benefit most from the introduction of LM to their clients and the actual financial benefits for the health care organizations were unclear. A lack of understanding about the purpose of the role of health care professionals in introducing LM to health care consumers hampers the implementation. As such, some described hesitance and even reluctance to inform their clients about LM. This experienced friction between the different financial gains of introduction of LM to clients by an health care organization was described by a manager as follows:

*“But you have to, it has to be really clear what that added value is for that client, because otherwise it’s like yes, but we are not commercial sellers, that is of course the first thing our professional said. They get a product, so why should I offer it, why, are they going to earn money off the backs of our clients?”*

(respondent 14, health care manager).

- *Lacking opportunities for flexibility of the health care system—barrier*

Some of the participants described that a different design of the health care system is necessary in order for health care professionals to have the opportunity to anticipate the notifications of LM by provision of the required care according to these LM notifications. In order to be able to react on LM notifications, flexibility in care is needed, resulting in new tasks and responsibilities. As an example, an informal caregiver said that if LM is telling a client has had a bad night of sleeping, it would be helpful if the health care professional has the flexibility to visit the patient at a different time than planned.

*“So they cannot say: ‘this client slept badly, so let this client sleep a little longer and come a little later, that’s really not possible. It is much too rigid for that, it is not flexible, such an organization.”*

(respondent 3, informal caregiver).

### 3.4. Reflection on NPT Construct Cognitive Participation 

A barrier for implementation among all stakeholders was found in terms of transition of care and responsibilities. Possibilities for a responsibility shift from HCP to IC, for example, in the desired follow up of LM, was lacking and was perceived to be important for LM to redeem its effects. A passive attitude to LM by health care professionals was found, caused by lack of awareness of LM by health care professionals and different perspectives about whether technology is part of the job of a health care professional. As such, a fit of the needed way of working for LM to redeem its intended effects was not present according to this construct.

- *Lacking opportunities in transition of care and responsibilities—barrier*

According to the majority of the respondents in this study, the informal caregiver was the key user of the LM system. It was expected that the informal caregiver would respond to alerts, created by the system, and should take an active role in transferring health status knowledge (acquired via the system) to the health care professional. 

Some health care professionals were unsure about what taking part in LM would entail for the informal caregiver. First, they were concerned that the informal caregiver would experience increased stress levels or burden. Second, they were concerned about the financial costs—specifically, that the use of LM would require older adults to enter into an Internet subscription.

Some health care professionals were therefore reluctant to introduce LM to the informal caregivers, as they perceived that LM could increase the burden of the informal caregiver. Informal caregivers, however, did not experience the burden of LM. They needed to get used to the notifications, but it was not felt to be a burden of LM.

The three health care organizations had different views on roles and responsibilities. One health care manager reflected on the fact that informal caregivers were part of the health care system of their clients.

*“The moment you hand it over to the health care provider, then you take it away from the informal caregiver. What we all really want is that the management of health remains as close as possible to our own client and to his or her own system. And so you have to ask yourself, is it wise to get him out of there? Because then you remove a part of the self-regulation.”*

(respondent 7, health care manager).

### 3.5. Reflection on NPT Construct Collective Action 

In this construct, we found barriers with respect to implementing LM in practice. All stakeholders perceived a lacking eligibility strategy for clients, as there were different ideas about a proper strategy by whom and when to introduce LM to clients and who was the actual target group of clients (eligibility). There were also different ambassador strategies that HCPs used in the three health care organizations. Lacking such strategies are perceived barriers for implementation in practice. As such, a structured way of working with LM in practice was not present according to this construct.

- *Unclear eligibility strategy for clients—barrier*

A triage system was lacking among all health care organizations about when to introduce LM and to whom. All stakeholders found this kind of strategy necessary to decide which clients could benefit from an introduction of LM. Stakeholders agree that clients in an early stage of dementia would be a good target group for LM because this group does not need a lot of care and LM could work in a supportive and preventative manner because of the ability to monitor the client. Clients who already are provided with a great amount of care are used to that way of working, and for them, there is less added value of LM as an extra service to the daily care that they already receive. In this study, the information sent by post from the municipality about LM was not always read by the informal caregiver. When clients got information by the involved health care organization without an informal caregiver during this introduction, for a client, the introduction was difficult to follow. The right moment to introduce LM to eligible clients was perceived to be before there was an actual need for professional care, and when informal caregiver and client were at the same time able to join the introduction of LM.

*“..there were mainly Social Support Law clients, so actually clients who did not really need a lot of care yet, so actually clients who did not really need a lot of care yet, but those where you could do a lot preventively. And we all have clients who are already in care and they are actually already used to a certain way of care; why would they use LM.”*

(respondent 14, health care manager).

*“It is important to be able to offer a little more preventively for people who have mild dementia. And things have to be deployed urgently more and more quickly and that is not possible at all unless we as health care professionals have to get a better picture preventively.”*

(respondent 12, health care provider).

In addition, vulnerable clients who received aid in housekeeping would not be considered as part of the target group for reasons of loneliness and depression. They need to be monitored by people instead of LM in order to reduce the risk of loneliness and depression.

*“And then you see that there is a lot of loneliness and aspects of depression, being vulnerable, not knowing how to handle but not actively acting on that, but just wait.”*

(respondent 1, health care manager).

Age sometimes plays a role and the level of social control of family or neighbors. Overall, younger informal caregivers were more keen to use LM, and people with a larger social network were less interested to use LM.

- *Unclear ambassador strategy (for HCP’s)—barrier*

In this study, we found that each health care organization decided different on the ambassador’s role of LM; there was no official choice made about introducing LM, following upon the decision of the client whether to start with LM after the introduction and the right person who was responsible for this task. In one health care organization people from the daily household were assigned to signalize who needed to get an introduction about LM but were perceived not to be the best choice for giving such introductions. In another health care organization, health care professionals got the task to introduce LM; however, the introduction then often came too late as clients then needed professional care instead of monitoring of their daily lives to prevent a care situation. Suggestions were made that introduction of LM by the Dutch Social Support Law or the general physician (GP) would be beneficial, as clients receiving support by this law or when clients are seen by a GP, are mostly not in direct need of daily care and thus a good starting point for introduction of LM.

### 3.6. Reflection on NPT Construct Reflexive Monitoring 

There was no systematic evaluation of the implementation of LM or the technology aspects of LM, organized by the LM supplier nor the health care organization, nor the informal caregivers, also no follow-up of the decision to start with LM of health care consumers and their informal caregivers was done by health care professionals. Therefore, we found limited results on the construct reflexive monitoring in terms of embedding LM in practice. What we did find were suggestions for improvement of the technology.

- *Lacking perceived reliability of the technology—barrier*

A few suggestions for improvement were addressed after using LM relating to the appearance of errors. The reliability of the LM was lacking at a few points, which made it difficult to trust LM in its use. For example, when a message about the inactivity of a client was provided, it was unclear where a client at that moment was present, and what the reason was of the inactivity, e.g., the client could sleep at that moment but also could have fallen.

*So that’s why we never know where she sleeps. She is inactive so they say, she is sleeping, but then she is not in bed at all. I find that unclear.”*

(respondent 11, informal caregiver).

## 4. Discussion

The purpose of our study was to understand factors that drive or impede successful implementation of LM for vulnerable older adults, using infrared sensors to record movements, as seen important from a multiple stakeholder. The study was based on the four constructs of the “NPT model” representing coherence, cognitive participation, collective action, and reflexive monitoring. It was found that stakeholders considered different aspects to be relevant within these constructs and thus did not always align with each other within the constructs of NPT. NPT offers a coherent set of explanations of implementation processes. The mechanisms that motivate these processes are characterized by NPT and can be used to understand implementation processes themselves. To succeed in implementation, it seems to be important that different stakeholders’ perspectives are transparent and that reflexive monitoring will take place with all involved stakeholders in order to obtain a mutual understanding of the needs and possibilities for implementation to be successful.

- Interpretation of findings:

We found experienced drivers according to the construct coherence and only barriers according to the constructs cognitive participation, collective action and reflexive monitoring. This study investigated the perceived importance of including the informal caregiver in the communication process as important aspect of successful implementation (NPT construct cognitive participation). Targeting the informal caregiver directly with information about LM is important as the informal caregiver is able to monitor whether there is a need for LM as a primary user of the LM system. In addition, informal caregivers’ opinion toward technology may also affect the client decision whether to start and continue to use the technology [27,28]. To give the informal caregiver a central role in the implementation and follow-up of LM, a more clear business case will be created. A clear business case is important for the understanding of and engaging with the intervention by health care managers that could influence an implementation success [29]. Clear role descriptions, implementation strategies, and aligning LM to organizational processes were lacking due to a push of the municipality to implement LM. Efficient management of this process is crucial [30]. In addition to organizational processes, care provision flexibility in response to LM was found to be lacking. Health care professionals stated that if they are facilitated in the opportunity to work in a flexible manner using LM, it would be beneficial for health care organizations. The working hours of the health care professionals are ideally used to what a client needs according to notifications of LM and not as in the current situation standardized once a week. In this way, working hours are used more efficiently.

The lacking support in role description reflected in the construct coherence (perceived value of the role of the health care organization in the light of cost efficiency), the construct cognitive participation (unclear responsibilities), and the construct collective action (unclear strategies) by, e.g., a proper training available and opportunities for flexibility in the health care system could have been discussed during evaluations with all involved stakeholders. However, during the implementation of LM, opportunities for ongoing review and feedback for involved stakeholders and, ideally, combined with technical stakeholders (supplier of LM) was lacking. This evaluation seems to be important for improving collaboration among stakeholders and reconfiguring roles and adjustments in distribution of tasks and processed necessary to reach the ultimate potential of the implemented technology [31]. 

For successful implementation of LM in vulnerable older adults living alone, it should also be clear what the perceived value is of LM, and for whom, as reflected in the construct coherence (values toward LM) and in the construct collective action (unclear strategies). The results made clear that LM could generate even more added value when introduced as preventive care, at a time when the user is not experiencing severe health problems yet. Consequently, LM should be introduced to the older vulnerable population in an early stage.

However, in this study, health care professionals were the ambassadors, and a clear instruction for them how to introduce LM was lacking. When distributing new technologies, the literature stresses the importance of sufficient staff availability, and regular staff training. Certain staff characteristics such as insecurity about technological or ethical issues have also been reported to impede implementation [32]. In addition to increased technology awareness, a multi actor approach could also help to disseminate information. Information provided by the health care professional or informal caregiver could be a strategy to introduce LM. Information could, for example, be provided by medical supply stores, general practitioners, governments, and health care insurance companies as well [33].

- Strengths and Limitations:

A strength of this study is the inclusion of directly involved stakeholders of three different health care organizations to support triangulation. The relative low number of inclusions are a limitation to generalize the results; however, we investigated the same perceived shift in roles between formal and informal caregivers in the three different health care organizations and we found several topics relevant for all stakeholders among the three health care organizations, which could be seen as support for the ecological validity of our study. Because LM was offered for free, cost-related factors that could play among vulnerable older adults to use a new technology were absent in this study [34]. As a result, pure stakeholders’ experiences in the care context with the implementation of LM for vulnerable older adults as well as stakeholders’ reflection of what is needed in order to be able to work with LM and their role in this implementation were studied. Additionally, the “NPT” was used as a conceptual framework allowing to map drivers and barriers of implementing LM in a systematic way. Using NPT increased our understanding about how stakeholders individually saw LM and in relation to teamwork (coherence), how stakeholders collectively bought into the new model of care (cognitive participation), how stakeholders put LM into action (collective action), and how stakeholders appraised it (reflexive monitoring). Although the stakeholders in the care context are the key figures in implementation, in complex implementation, not only stakeholders in the care context play a role. Our study is limited by the fact that it did not include other stakeholders in the wider system, for example, the service providers, to get the broader picture of implementation. In this study, implementation-related factors related to the perspectives of stakeholders in the care context were clear, while perspectives on the boundary between, for instance, the supply side (developer) and demand side (user) were not clear. From theoretical frameworks such as NASSS, we know that adoption of technology across multiple NASSS domains, covering more than only the care context of implementation, is important. The NASSS framework explains difficulties in adoption of technology by stakeholders and the scale-up, spread, and sustainability of the technology in all contexts. Since failure of implementation is often linked to complexity across multiple NASSS domains and its contexts, it is important to investigate the complexity within all domains of NASSS to account for all levels of complexity in order to know how to reduce this complexity with the aim to improve the implementation of a new technology [35].

Furthermore, we did not investigate whether the assumed mutual roles in implementation of different stakeholders were the same. For example, our research shows that the health care professional wants the informal caregiver to act on the messages of LM. However, we do not know what the informal caregiver thinks of this role, and we also do not know what the client in this situation wants. In the context of research, this information could be obtained during a focus group in order to reach transparency of roles among all involved stakeholders and possibly to reach common goals for successful implementation. In practice, structured meetings or sessions where all stakeholders have the opportunity to discuss the implementation and looking for improvements would be helpful and were missing in this study.

One could argue that implementation evaluations covering a longer period of time are needed to determine how dynamics between stakeholders influence the effective provisioning of LM that can help vulnerable older adults to age in place. As a limitation of our study, we did not strive for data saturation. This means that our results cannot be generalized to each situation where LM is implemented. However, our results could be used as a starting point to improve the implementation of LM for vulnerable older adults. In this study, the financial barrier was excluded by the municipality; however, it did not lead to successful implementation.

- Implications for research and practice:

Our results indicate that a prerequisite for implementing LM is creating sufficient transparency on the different roles and responsibilities stakeholders have in working with LM and to align on this when perspectives are different. Our recommendations for further research are first to bring different stakeholders’ perspectives together for this and other technology that is used to help vulnerable older adults remain independent at home. A focus group including perspectives from stakeholders from the care process as well as perspectives form the supply side—more specifically, the actual installers of the technology—will be the recommended methodology. This is important for stakeholders to understand each other’s perspectives and to reach new ways of collaboration in care processes, including, based on our study, required changes in care structures and responsibilities due to increased importance of the informal caregiver. In addition, knowledge is required to prepare (in)formal caregivers for implementing and using technology such as LM. There is a need for technology introduction in a way that older adults and (in)formal caregivers are enabled to make shared decisions [36]. Regarding implementation, there is a need to take account of the wider institutional and sociocultural context next to the value of the user in order to be able to fully spread and sustainable technology [37]. Third, NPT has up until now been used within health care organizations to clarify normalization of technology use by health care professionals. In our study, we found that it also can be used in the evaluation of technology use by other users such as informal caregivers. This knowledge could be used as first steps to better implement LM within formal and informal care, which may result in better supporting of mental health in vulnerable older adults living alone at home.

## 5. Conclusions

This study highlights the complex nature of implementing LM and suggests the need for transparency of stakeholders’ perspectives about new ways of collaboration in supporting living alone at home for older persons, in which the role of the informal caregiver and health care professional system needs to be clear. In addition, the LM system itself needs to include the new ways of organizing health care in the future in order to be effective. There is also a need for clarity of the eligibility of potential users and of the involved ambassadors in the introduction of LM. Last, reflection on the new situation by involved stakeholders after introduction should be part of the process of implementation.

## Figures and Tables

**Table 1:** Study population.

	Role ^1–3^	Gender (Male/Female)	Age (Years)
Respondent 1	3	Male	55
Respondent 3	1	Female	54
Respondent 4	3	Female	51
Respondent 5	1	Male	69
Respondent 7	3	Female	62
Respondent 8	1	Female	45
Respondent 9	2	Female	51
Respondent 10	2	Female	45
Respondent 11	1	Female	65
Respondent 11	1	Female	54
Respondent 12	2	Female	32
Respondent 13	2	Female	34
Respondent 14	3	Female	49
Respondent 15	3	Male	52

^1^ Informal caregiver, ^2^ health care professional, ^3^ health care manager.

## Appendix A

**Table A1:** Interview guide according to NPT.

	Question
Main questions	1—How did you get in touch with LM?
	2—Can you describe what it means to use LM?
	3—How do you experience the use of M?
	4—What are the benefits of using LM?
	5—What are the disadvantages of using LM?
	6—How can LM be improved?
	7—Which parts or actions of LM would you like to change? Why?
Construct: coherence	1—How did the care you provide using LM differ from the care you provided without the use of LM?
	2—What was the added value of using LM for the care of your client/relative in your opinion?
	3—For a system like LM it is important that people involved, work together towards the same goal. How did you perceive that?
	4—Did LM correspond to your definition of good (informal/professional) care? Did LM correspond to the organizations’ definition of good care?
Construct: cognitive participation	1—How do you or involved people (family or team members) perceive the usefulness of LM?
	2—Do you think that all involved people experienced confidence in the use of LM?
	3—Did all involved people support the use of LM? In what way was LM supported/not supported?
	4—Were people involved willing to invest time and energy in the use of LM?
	5—Was it clear to all involved people (family or team members) how the use of LM affects their daily tasks and responsibilities?
	6—Did you feel responsibility for acting according to notifications of LM?
	7—What is your opinion about a training to be able to use LM?
Construct: collective action	1—Have you been able to work successfully with LM? If so, how come, if not, what do you need?
	2—What is important with respect to training, in your opinion?
	3—Does the organization you are employed at, encourage the use of LM?
	4—Does the use of LM fit to your way of working?
	5—What influence did LM have on the responsibilities for the different departments within the care for the older adult?
Construct: reflexive monitoring	1—What are the advantages and disadvantages of using LM for those involved?
	2—Was it clear or will it become clear what the effect was of LM?
	3—Did you feel possibilities to evaluate LM/to provide feedback during the use of LM?
	4—Did you have the ability to make adjustments in the use of LM after gaining experience with LM?

NPT = Normalization Process Theory.

## References

[1] Pickard L. (2015). A growing care gap? The supply of unpaid care for older people by their adult children in England to 2032. Ageing Soc..

[2] Grande G., Qiu C., Fratiglioni L. (2020). Prevention of dementia in an ageing world: Evidence and biological rationale. Ageing Res. Rev..

[3] World Health Organization (2019). Risk Reduction of Cognitive Decline and Dementia: WHO Guidelines.

[4] Lorenz K., Freddolino P.P., Comas-Herrera A., Knapp M., Damant J. (2019). Technology-based tools and services for people with dementia and carers: Mapping technology onto the dementia care pathway. Dementia.

[5] Holthe T., Halvorsrud L., Karterud D., Hoel K.-A., Lund A. (2018). Usability and acceptability of technology for community-dwelling older adults with mild cognitive impairment and dementia: A systematic literature review. Clin. Interv. Aging.

[6] Astell A.J., Bouranis N., Hoey J., Lindauer A., Mihailidis A., Nugent C., Robillard J. (2019). Technology and Dementia Professional Interest Area. Technology and Dementia: The Future is now. Dement. Geriatr. Cogn. Disord..

[7] Gibson G., Newton L., Pritchard G., Finch T., Brittain K., Robinson L. (2016). The provision of assistive technology products and services for people with dementia in the United Kingdom. Dementia.

[8] Urwyler P., Stucki R., Rampa L., Müri R., Mosimann U.P., Nef T. (2017). Cognitive impairment categorized in community-dwelling older adults with and without dementia using in-home sensors that recognise activities of daily living. Sci. Rep..

[9] Nakaoku Y., Ogata S., Murata S., Nishimori M., Ihara M., Iihara K., Takegami M., Nishimura K. (2021). AI-Assisted In-House Power Monitoring for the Detection of Cognitive Impairment in Older Adults. Sensors.

[10] Wichert R., Furfari F., Kung A., Tazari M.R., Wichert R., Eberhardt B. (2012). How to Overcome the Market Entrance Barrier and Achieve the Market Breakthrough in AAL. Proceedings of the Ambient Assisted Living: 5 AAL-Kongress 2012.

[11] Chung J., Reeder B., Lazar A., Joe J., Demiris G., Thompson H.J. (2014). Exploring an informed decision-making framework using in-home sensors: Older adults’ perceptions. J. Innov. Health Inform..

[12] Campling N., Pitts D.G., Knight P.V., Aspinall R. (2017). A qualitative analysis of the effectiveness of telehealthcare devices (ii) barriers to uptake of telehealthcare devices. BMC Health Serv. Res..

[13] Kruse C., Fohn J., Wilson N., Patlan E.N., Zipp S., Mileski M. (2020). Utilization Barriers and Medical Outcomes Commensurate with the Use of Telehealth among Older Adults: Systematic Review. JMIR Med. Inform..

[14] Karlsen C., Ludvigsen M.S., Moe C.E., Haraldstad K., Thygesen E. (2017). Experiences of the home-dwelling elderly in the use of telecare in home care services: A qualitative systematic review protocol. JBI Database Syst. Rev. Implement. Rep..

[15] Robinson E.L., Park G., Lane K., Skubic M., Rantz M. (2020). Technology for Healthy Independent Living: Creating a Tailored In-Home Sensor System for Older Adults and Family Caregivers. J. Gerontol. Nurs..

[16] Klemets J., Määttälä J., Hakala I. (2019). Integration of an in-home monitoring system into home care nurses’ workflow: A case study. Int. J. Med. Inform..

[17] Wade V.A., Taylor A.D., Kidd M.R., Carati C. (2016). Transitioning a home telehealth project into a sustainable, large-scale service: A qualitative study. BMC Health Serv. Res..

[18] Schreiweis B., Pobiruchin M., Strotbaum V., Suleder J., Wiesner M., Bergh B. (2019). Barriers and Facilitators to the Implementation of eHealth Services: Systematic Literature Analysis. J. Med. Internet Res..

[19] Dickinson C., Gibson G., Gotts Z., Stobbart L., Robinson L. (2017). Cognitive stimulation therapy in dementia care: Exploring the views and experiences of service providers on the barriers and facilitators to implementation in practice using Normalization Process Theory. Int. Psychogeriatr..

[20] May C. (2006). A rational model for assessing and evaluating complex interventions in health care. BMC Health Serv. Res..

[21] McEvoy R., Ballini L., Maltoni S., O’Donnell C.A., Mair F.S., MacFarlane A. (2014). A qualitative systematic review of studies using the normalization process theory to research implementation processes. Implement. Sci. IS.

[22] May C.R., Cummings A., Girling M., Bracher M., Mair F.S., May C.M., Murray E., Myall M., Rapley T., Finch T. (2018). Using Normalization Process Theory in feasibility studies and process evaluations of complex healthcare interventions: A systematic review. Implement. Sci. IS.

[23] Valaitis R., Cleghorn L., Dolovich L., Agarwal G., Gaber J., Mangin D., Oliver D., Parascandalo F., Ploeg J., Risdon C. (2020). Examining Interprofessional teams structures and processes in the implementation of a primary care intervention (Health TAPESTRY) for older adults using normalization process theory. BMC Fam. Pract..

[24] Dalkin S.M., Hardwick R.J.L., Haighton C.A., Finch T.L. (2021). Combining Realist approaches and Normalization Process Theory to understand implementation: A systematic review. Implement. Sci. Commun..

[25] Finch T.L., Mair F.S., O’Donnell C., Murray E., May C.R. (2012). From theory to ‘measurement’ in complex interventions: Methodological lessons from the development of an e-health normalisation instrument. BMC Med. Res. Methodol..

[26] Bradley E.H., Curry L.F., Devers K.J., Devers K.J. (2007). Qualitative data analysis for health services research: Developing taxonomy, themes, and theory. Health Serv. Res..

[27] Waller A., Dilworth S., Mansfield E., Sanson-Fisher R. (2017). Computer and telephone delivered interventions to support caregivers of people with dementia: A systematic review of research output and quality. BMC Geriatr..

[28] Guisado-Fernández E., Giunti G., Mackey L.M., Blake C., Caulfield B.M. (2019). Factors Influencing the Adoption of Smart Health Technologies for People with Dementia and Their Informal Caregivers: Scoping Review and Design Framework. JMIR Aging.

[29] Kelley R., Griffiths A.W., Shoesmith E., McDermid J., Couch E., Robinson O., Perfect D., Surr C.A. (2020). The influence of care home managers on the implementation of a complex intervention: Findings from the process evaluation of a randomised controlled trial of dementia care mapping. BMC Geriatr..

[30] Taylor A., Wade V., Morris G., Pech J., Rechter S., Kidd M., Carati C. (2016). Technology support to a telehealth in the home service: Qualitative observations. J. Telemed. Telecare.

[31] Kidholm K., Ekeland A.G., Jensen L.K., Rasmussen J., Pedersen C.D., Bowes A., Flottorp S.A., Bech M. (2012). A model for assessment of telemedicine applications: Mast. Int. J. Technol. Assess. Health Care.

[32] Christie H.L., Bartels S.L., Boots L.M., Tange H.J., Verhey F.R., de Vugt M.E. (2018). A systematic review on the implementation of eHealth interventions for informal caregivers of people with dementia. Internet Interv..

[33] Freiesleben S.D., Megges H., Herrmann C., Wessel L., Peters O. (2021). Overcoming barriers to the adoption of locating technologies in dementia care: A multi-stakeholder focus group study. BMC Geriatr..

[34] Kim E., Gellis Z.D., Bradway C., Kenaley B. (2019). Key Determinants to using telehealth technology to serve medically ill and depressed homebound older adults. J. Gerontol. Soc. Work..

[35] Greenhalgh T., Wherton J., Papoutsi C., Lynch J., Hughes G., A’Court C., Hinder S., Procter R., Shaw S. (2018). Analysing the role of complexity in explaining the fortunes of technology programmes: Empirical application of the NASSS framework. BMC Med..

[36] Wrede C.A.-O., Braakman-Jansen A.A.-O., van Gemert-Pijnen L.A.-O. (2021). Requirements for Unobtrusive Monitoring to Support Home-Based Dementia Care: Qualitative Study among Formal and Informal Caregivers. JMIR Aging.

[37] Greenhalgh T., Wherton J., Papoutsi C., Lynch J., Hughes G., A’Court C., Hinder S., Fahy N., Procter R., Shaw S. (2017). Beyond Adoption: A New Framework for Theorizing and Evaluating Nonadoption, Abandonment, and Challenges to the Scale-Up, Spread, and Sustainability of Health and Care Technologies. J. Med. Internet Res..

